# Design of a low-cost microphone array for portable multi-platform applications

**DOI:** 10.1016/j.ohx.2025.e00710

**Published:** 2025-10-01

**Authors:** Luis Corral, Pablo E. Román

**Affiliations:** Universidad de Santiago de Chile, Departamento de Ingeniería Informática, Santiago, Chile

**Keywords:** Acoustic camera, Holography, Point cloud

## Abstract

Acoustic imaging analysis of noise sources is a widespread method to obtain source spatial positioning and parameters like sound pressure. The challenge is to record audio from multiple microphones simultaneously and optionally capture color images from a camera for final overlapped sound localization display. We propose a multiple hardware component interconnection device, with necessary and optional parts available from different retailers and minimal printed circuit board designs. The setup is based on a 3D-printed base that can be modified to achieve different portable microphone array configurations. The final product is USB 2.0 compliant and can be connected to multiple computers and operating system, as well as various development boards. The simple script provided allows recording 16-channel files at 48 kHz and 32 bits, alongside optional colored point clouds from a depth camera. Measurements taken in an anechoic chamber and post-processing with inverse methods result in holographic reconstruction of noise sources surface parameters and a sound power approximation.

## Highlights

• The microphone array is a USB 2.0 audio device for Linux, Windows, and macOS.
• Provided design files can be modified to study new array configurations.
• With optional components the device is a low-cost portable acoustic camera.

## Graphical abstract

**Figure:**
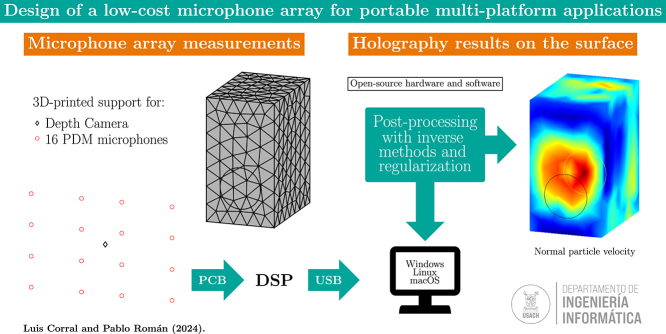

**Table:** 

Specifications table	
Hardware name	Multi-platform microphone array
Subject area	•Engineering and material science
Hardware type	•Imaging tools • Measuring physical properties and in-lab sensors
Closest commercial analog	miniDSP UMA-16 v2 USB mic array
Open source license	CERN Open Hardware Licence Version 2 – Strongly Reciprocal
Cost of hardware	$ 178 USD necessary components, $ 688 USD optional components
Source file repository	https://doi.org/10.5281/zenodo.13353428

## 1 Hardware in context

Undesirable noise levels are present in every industry, in activities such as leisure or transportation, and in many others common to modern societies. It is especially important to comprehensively understand and study this problem to improve mitigation strategies especially because it is a widespread issue with significant impact on everyday life. The principal motivation to develop noise control strategies is the harmful effects that high sound pressure levels can have on the receivers. This includes insomnia, headaches, and an increased risk of coronary artery disease when exposed to environmental noise, while in extreme cases (such as occupational noise) it may cause mild to severe hearing loss [1], [2]. Typical assessment techniques involve taking pressure, intensity, or laser vibrometry measurements of sources in controlled environments such as anechoic chambers. Apart from laser vibrometry, these evaluations typically result in third-octave pressure or sound power values. Thanks to the latest technological advances, it is possible to construct devices such as acoustic cameras equipped with multiple microphones. Such devices provide more detailed information about noise sources, such as location and surface distribution, in addition to the third-octave parameters mentioned earlier [3].

The term microphone array is used to define a set of sound sensors arranged in specific spatial configurations. Microphone arrangements can take the form of a line (one dimension), a plane (two dimensions), or a cube/sphere (three dimensions). A historical review of the theory, applications, and post-processing techniques for microphone arrays can be found in comprehensive reviews by P. A. Nelson [4] and R. Merino-Martinez [5]. The latest developments in this field include beamforming algorithms in two and three dimensions, which benefit from deconvolution methods to focus the source localization spatial position. Other techniques known as inverse methods [6], can be used to extract surface holography and different sound parameters. Nowadays, acoustic cameras are available from a wide variety of commercial retailers [7] in various forms and with different application software. The price range of currently available acoustic cameras can reach several thousand USD. Different spatial configurations of microphone arrays offer advantages in frequency range and accuracy [8], an area of study that is continually evolving. This has led us to design and construct a low-cost device compatible with multiple operating systems and platforms. The microphone positions in our device can be easily changed using the simple three-dimensional geometry provided, that can be exported in three parts for 3D printing and later assembled with ease. We hope that this product will be useful for the study and teaching of noise propagation and microphone array techniques.

## 2 Hardware description

**Fig. 1:**
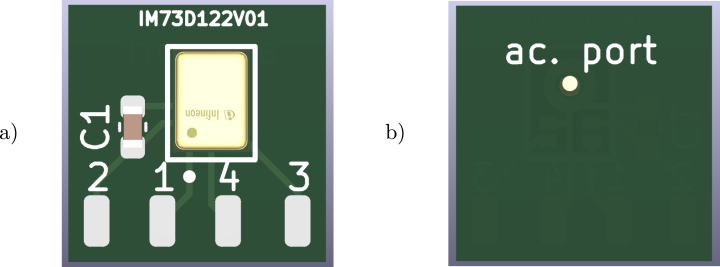
PDM microphones PCB, (a) front and (b) back.

Our design consists of 16 micro-electro-mechanical system (MEMS) microphones soldered onto a custom printed circuit board (PCB) mounted on a 3D-printed support. The selected device [9] can be replaced with other bottom-port PDM microphones (with the same supply voltage) and the provided PCB schematic (see Fig. 1) can be modified to accommodate such substitution. This PCB includes four solder pads for a flat ribbon cable connection to the PDM microphone pins: 2 for 3.3 V supply (with a 0.1 μF bypass capacitor), 1 for the PDM data signal, 4 for ground, and 3 for the digital clock signal.

**Fig. 2:**
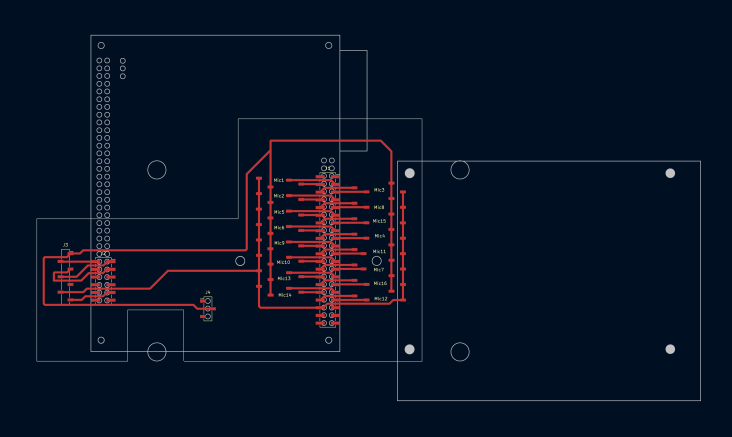
Mounting PCB with the DSP and Jetson Nano boards.

**Fig. 3:**
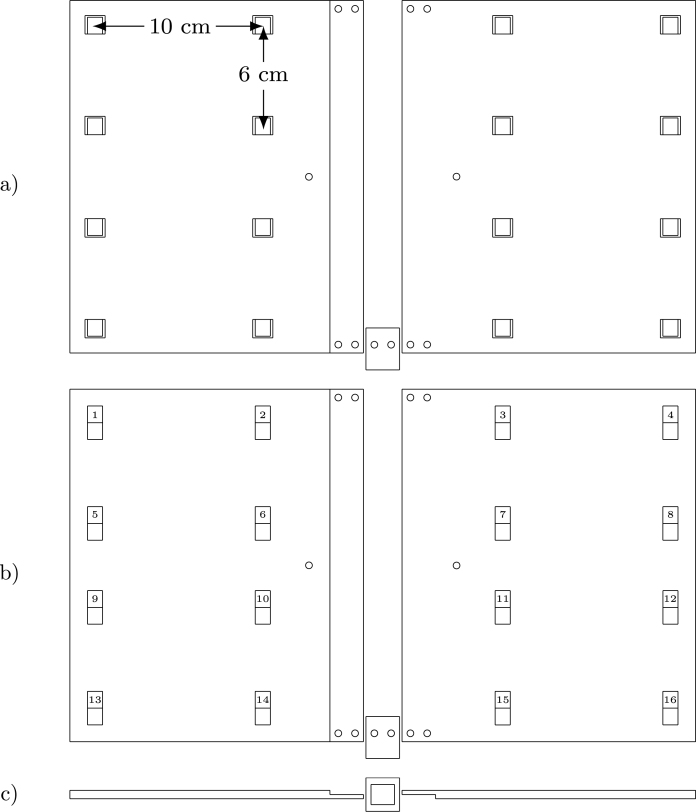
3D printable design, (a) front, (b) back and (c) bottom.

The entire device is based on a 3D-printed support (see Fig. 3). It is designed in three parts to fit within a 22 × 22 cm surface area 3D printer. The holes in the main surface include front pockets for the microphone’s PCB placement and back pockets for flat ribbon cable output. The fully assembled plate measures 33 × 22 cm, and holes are provided for M3 screws and fasteners. The bottom support contains a hole for a 1/4-20 UNC nut for camera tripod mounting. All pins of the PDM microphone are connected to a digital signal processing (DSP) board [10] that provides a USB 2.0–compliant audio device to a Linux (tested on Ubuntu 22.04.3 LTS and Jetson Linux 36.3), Windows (tested on Windows 11), or macOS (though untested on this OS) host. Since the PDM and DSP USB audio signals require a high-quality digital clock, a board based on the Si5351A chip (such as this one from Adafruit^1^) is used, programmed via I^2^C on the DSP. Several mounting options for the DSP are available, such as flat ribbon header connectors and plastic spacer/feet PCB mounting. However, we provide a simple, single-sided mounting PCB based on surface-mounted device (SMD) header connectors, which allow easy assembly of all necessary and optional hardware components (see Fig. 2). Two M3 holes on the mounting PCB and the 3D-printed support allow everything to be fixed together, and an Intel RealSense d435i [11] optional depth camera can be added in this stage. The second optional component integrates the system, as an NVIDIA Jetson Nano Developer Kit [12] provides USB 3.0 ports to connect the RealSense camera and the DSP. The third optional component is a PCIe M.2 Wi-Fi/Bluetooth adapter for the Jetson Nano. To power everything, a USB Type-C power delivery board (such as this one from Adafruit^2^) is used for the Jetson Nano via barrel jack and the DSP via micro-USB.

The main characteristics of the proposed design are:

• The microphone array is a plug-and-play USB 2.0 compliant audio device for Linux, Windows, and macOS.
• Provided CAD files for 3D printing and PCB designs can be modified to study new MEMS microphones or array configurations.
• The optional development board and depth camera addition results in a low-cost portable acoustic camera.

## 3 Design files summary

**Table:** 

Design filename	File type	Open source license	Location of the file

3dprintedsupport.FCStd	3D printing file	CERN Open Hardware Licence Version 2	Available with the article
microphonepcb.kicad_pcb	PCB file	CERN Open Hardware Licence Version 2	Available with the article
mountingpcb.kicad_pcb	PCB file	CERN Open Hardware Licence Version 2	Available with the article
dspfirmware.xe	DSP firmware	CERN Open Hardware Licence Version 2	Available with the article
BOM.csv	BOM file	CERN Open Hardware Licence Version 2	Available with the article

3dprintedsupport.FCStd: Main support designed in FreeCAD software for 3D printing. It is created in three parts that can be exported in stereolithography (STL) format for printing.

microphonepcb.kicad_pcb: Microphone PCB board designed in KiCAD software. Includes the microphone footprint, and the 3D design^3^ (only necessary for viewing) can be found online.

mountingpcb.kicad_pcb: Mounting PCB board designed in KiCAD software with SMD header connectors.

dspfirmware.xe: Firmware executable file for XMOS xTimeComposer software flash of the DSP board.

BOM.csv: Complete and detailed bill of materials (BOM) as a comma-separated values (CSV) file.

## 4 Bill of materials summary

The price and material sources for the main necessary components are listed below. A detailed list is included as a CSV file. Shipping costs and soldering supplies are not included in the bill of materials.

**Table:** 

Designator	Component	Number	Cost per unit - currency	Total cost - currency	Source of materials	Material type

3D printed support	3dprintedsupport	1	23.92 USD	23.92 USD	Orballo Printing	Polymer
PDM microphone	448-IM73D122V01 XTMA1CT-ND	16	1.898 USD	30.368 USD	DigiKey	Semiconductor
Microphone PCB	microphonepcb	16	0.25 USD	5 USD	PCBWay	Composite
Flat Ribbon Cable	3M156838-1-ND	16	0.429 USD	6.864 USD	DigiKey	Composite
Mounting PCB	mountingpcb	1	5 USD	5 USD	PCBWay	Composite
Digital clock	1528-1206-ND	1	7.95 USD	7.95 USD	DigiKey	Composite
USB Type-C power	1528-5807-ND	1	5.95 USD	5.95 USD	DigiKey	Composite

The cost of the 3D printed base design is calculated by considering material, labor, and equipment expenses. PCB prices are estimated from similar project fabrication quotes without assembly costs. Details of the optional components are listed below.

**Table:** 

Designator	Component	Number	Cost per unit - currency	Total cost - currency	Source of materials	Material type

Jetson Nano	102110417	1	149 USD	149 USD	Seeed Studio	Composite
Intel RealSense	2311-82635D435ID K5P-ND	1	355 USD	355 USD	DigiKey	Composite

## 5 Build instructions

**Fig. 4:**
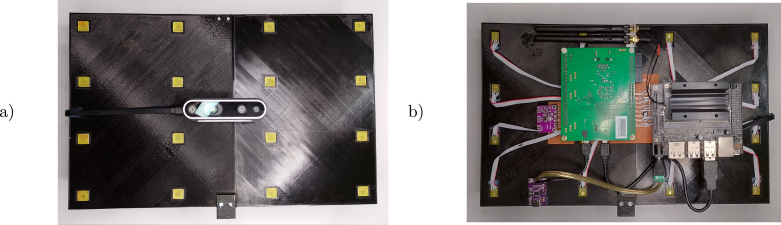
Final assembly, (a) front and (b) back.

*The assembly of the necessary parts is detailed below (see* Fig. 4 *):*

1. Export each body part (Left, Right, and Support) from the 3dprintedsupport.FCStd file to STL and print them. As a reference, we used an Ender3 3D printer with good results. Sanding and cyanoacrylate glue adhesion is recommended before nut and screw fixation.
2. If the microphone PCB is not yet assembled, a simple procedure is to place a PDM microphone and a bypass capacitor with solder paste and fuse them at 260${}^{\;\circ }$C with a reflow heat gun. Optionally, exact reflow oven temperature ramps are provided in the component’s datasheets.
3. Next, peel and fix the flat ribbon cable to the microphone PCB pins with a soldering iron. Repeat this process for all 16 microphone PCBs.
4. Use cyanoacrylate glue to fix all microphone PCBs in the support holes, positioning the flat ribbon cable at the back.
5. Attach metal spacers to the mounting PCB and screw it in place (considering the optional Intel RealSense camera) to the 3D printed support assembly.
6. Next, solder all the microphone flat ribbon cables and header connectors to the mounting PCB. A table with interconnection pins is provided in Appendix.
7. Attach and secure the DSP board to the mounting PCB J1 and J2 headers. Additional plastic spacers can be glued to the 3D printed support assembly to provide better support.
8. Attach the header pins to the digital clock board with a soldering iron and connect it to the J3 socket on the mounting PCB.
9. Glue plastic spacers to the 3D printed support assembly and secure the USB Type C power board in place.
10. Cut and strip the USB A end of the micro-USB DSP cable, solder the ends, and attach them to the USB Type C power board. Connect the micro-USB end to the J15 connector on the DSP board.

The optional component mounting is detailed next:

1. Secure the Jetson Nano to the mounting PCB with metal spacers. Additional plastic spacers can be glued to the 3D printed support assembly for better support.
2. Place the provided jumper on the Jetson Nano J48 (see the Jetson Nano manual [12]).
3. Cut, strip, and solder the ends of the Jetson Nano power cable. Attach the ends to the USB Type C power board and the DC barrel jack. Connect the DC barrel jack to the Jetson Nano.
4. Connect the DSP and the RealSense camera to the Jetson Nano via the provided USB cables.

## 6 Operation instructions

These instructions require an Ubuntu 22.04.3 LTS 64-bit computer. However, all the software used is fully compatible with Windows and macOS. Start by flashing the firmware on the DSP board.

1. Download the file dspfirmware.xe to your home  directory.
2. Open a terminal and install OpenJDK 8:

**Figure:**
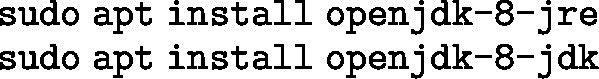
3. Download xTimeComposer 14.4.1^4^ to your home  directory (you will need a user account).
4. From your home  directory, extract the files via the terminal (check the file name):

**Figure:**
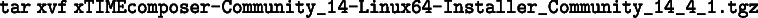
5. Navigate to the parent folder (check the folder names):

**Figure:**
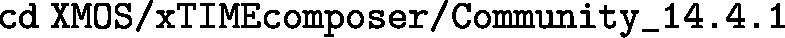
6. Connect the supplied xTAG v3.0 to the DSP board’s xSYS connector, and the micro-USB to an available USB port on your computer. Then run the following scripts to activate the USB driver:

**Figure:**
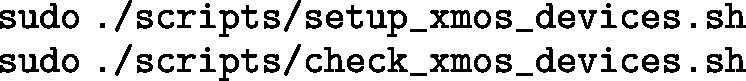
7. Check the connection of the xTAG v3.0:

**Figure:**
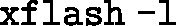
8. Flash the XMOS executable file dspfirmware.xe:

**Figure:**
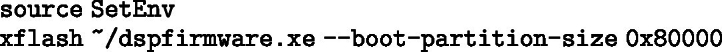

Next, set up the Jetson Nano for a fully portable system.

1. Follow the steps on the *Get Started with Jetson Nano Developer Kit*^5^ guide to write the SD card and boot the device for the first time. Ensure there is an internet connection and an IP address in the same range as your computer (a simple home router will work).
2. Download the recorder file recorder.cpp to your home  directory and send a copy to the Jetson Nano’s home  folder:

**Figure:**
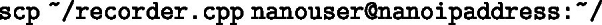
3. Connect to the Jetson Nano via SSH:

**Figure:**
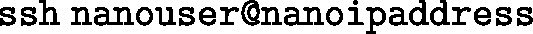
4. Once connected, install the RealSense SDK using the straightforward method.^6^
5. Compile the recorder file with the !nvcc CUDA compiler (gcc will work too):

**Figure:**
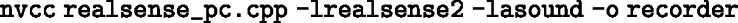
6. Run the recorder file; an audio WAV file and a point cloud file will be saved in the home directory (mic_array_audio.wav and realsense_pointcloud.ply files):

**Figure:**
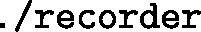
7. Back on your computer terminal, copy the files back with scp:

**Figure:**
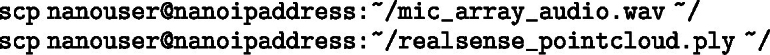
8. A MATLAB script for post-processing audio and point cloud files, post_processing.m, is included to obtain results.

## 7 Safety recommendations

Proper electronic systems laboratory safety guidance is recommended for the construction of the proposed device. In general, an ESD protection protocol must be implemented, lead-free solder and adequate ventilation must be ensured. A fire extinguisher and appropriate personal protective equipment (PPE) must be available at all times.^7^ Additional electronics safety recommendations include working on a hard, level surface, maintaining proper cable management and power practices, keeping away from heat sources and wet environments, and avoiding work during electrical storms.^8^

## 8 Validation and characterization

**Fig. 5:**
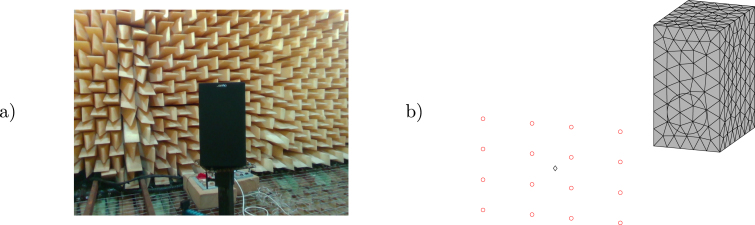
Loudspeaker in the anechoic chamber (a) and measurement diagram (b).

Test measurements took place in the anechoic chamber of the Acoustics Institute at the Universidad Austral de Chile. The objective of the first experiment was to measure a loudspeaker (see Fig. 5) with the microphone array and compare the obtained normal particle velocity $v_{s}^{n}$ to the cone’s velocity profile v(σ) (with σ being the distance to the center of the cone) obtained with an analytical solution based on Zernike polynomials [13] and pressure measurements taken on-axis (with a Class 1 Cirrus Research CR:171B sound level meter and its calibrator CR:515). The microphone array measurement was taken at 0.757325 m (calibrated with the RealSense camera), where a 3D model of the speaker with 301 surface nodes and 598 elements was positioned.

**Fig. 6:**
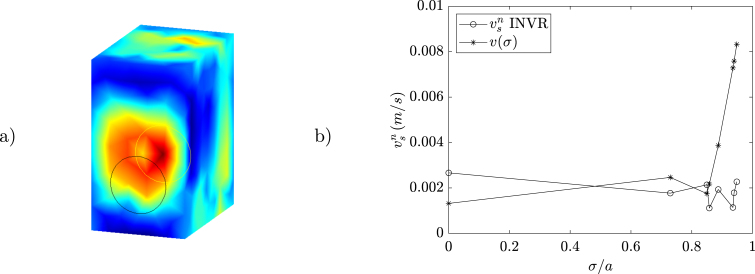
Surface holography INVR (a) and normal particle velocity (b).

**Fig. 7:**
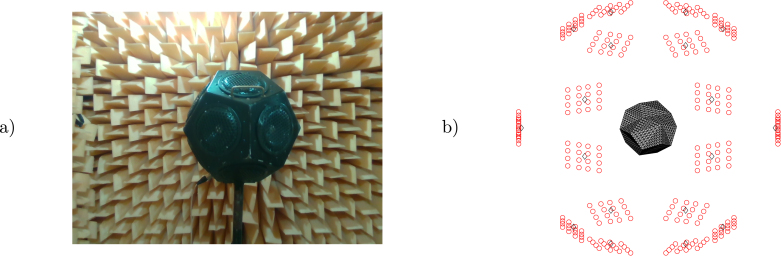
Omnidirectional source in the anechoic chamber (a) and measurement diagram (b).

The measurement scenario can be expressed as an inverse problem on the frequency domain in the form

(1) $$p_{h}=Hc\;\;\;$$

(2) $$v_{s}^{n}=Qc\;\;,$$

where the matrices H and Q can be obtained from the solution of the Helmholtz equation using different methodologies and $p_{h}$ are the pressure values from the microphone array in vector form. The formulation using the vector c allows the unification of the problem, which depends on the solution methodology. The vector c is equal to the normal particle velocity $v_{s}^{n}$ in methodologies based on numerical integration (such as the Equivalent Source Method, ESM, and the Boundary Element Method, BEM, where Q is the identity matrix) or is related to $v_{s}^{n}$ through the matrix Q (as in the Helmholtz Equation Least Squares, HELS). Results for 1605.4687 Hz, obtained with the analytical solution and with an inverse formulation based on the BEM and the radiation resistance matrix [14] (referred to as INVR) with the alternating direction method of multipliers error minimization [15] are shown in Fig. 6. While the maximum location of the cone’s center is not exact, the normal particle velocity values are close to the analytical solution. In the second experiment, a sound power measurement approximation based on ISO 3744 [16] (using 10 measurement points) of an omnidirectional noise source is compared with an estimate of the surface intensity integral of the sound pressure and normal particle velocity obtained with inverse methods. The microphone array measurements are taken surrounding the source (see Fig. 7) and the results are presented in IEC 61260 [17] third octave frequency bands. A 3D model of the source with 1154 surface nodes and 2304 elements was placed with the help of RealSense point cloud measurements.

**Fig. 8:**
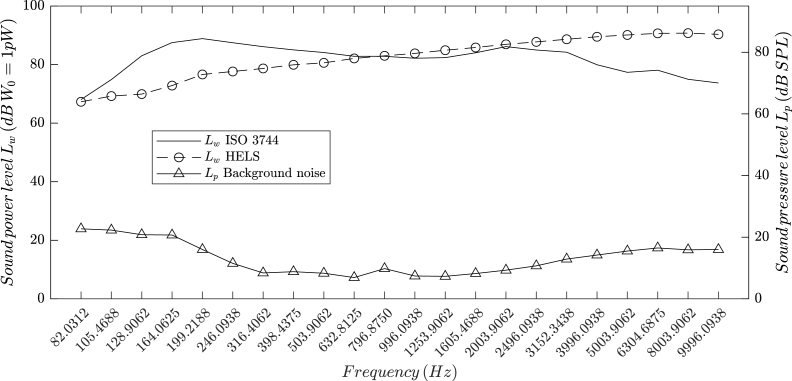
ISO 3744 and HELS sound power results.

**Fig. 9:**
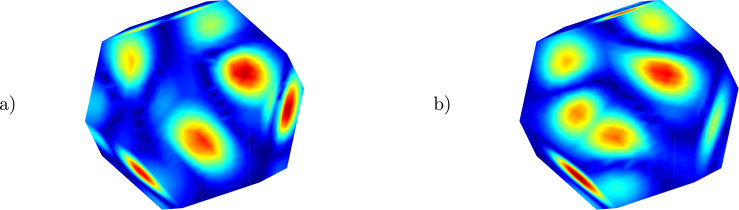
Surface holography for the normal particle velocity HELS 128.9062 Hz (a) and 316.4062 Hz (b).

**Fig. 10:**
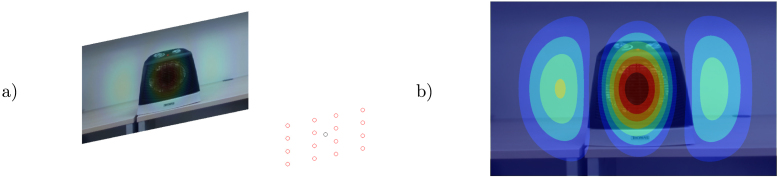
Holography image of the maximum particle velocity for the space heater at 90 Hz.

Sound power results using the ISO 3744 method and the Helmholtz equation least-squares (HELS) method [18] with damped singular value decomposition and L-curve regularization [19], [20] are shown in Fig. 8. Holography of the normal particle velocity on the surface at 128.9062 and 316.4062 Hz is presented in Fig. 9, where a fair approximation can be observed in both cases. All sound level meter pressure and microphone array measurements were taken while reproducing pink noise on the sources. The DSP board recorded audio WAV files at a 48 kHz sampling frequency and 32-bit depth in little-endian format. Post-processing involved applying an order-8 zero-phase band-pass filter from 1 to 22.05 kHz to suppress DC drift and undesired high-pitched noise, with all reconstruction and reporting limited to 10 kHz. This choice aligns with common environmental and industrial modeling standards (for example, ISO 9613 or VDI 3760), in which the sound power measurement results of this low-cost device may be used as input noise source data. Retaining full-band processing is still useful for diagnostics and future extensions, but all figures and metrics are intentionally reported up to 10 kHz. Filtering is followed by a short-time Fourier transform using a 4096-sample Hann window without overlap to reduce compute/memory load in order to obtain frequency-domain complex pressure values. Fig. 10 shows results of measurement operation under typical room conditions. We measured a space-heater fan at 60 cm on axis, where reflections and background noise are present. The holography maximum at the fan-blade center at 90 Hz confirms reliable source localization despite reflections and noise effects.

## CRediT authorship contribution statement

**Luis Corral:** Writing – original draft, Software, Resources, Investigation, Funding acquisition, Formal analysis, Data curation, Conceptualization. **Pablo E. Román:** Writing – review & editing, Validation, Supervision, Project administration, Methodology, Conceptualization.

## Declaration of competing interest

The authors declare that they have no known competing financial interests or personal relationships that could have appeared to influence the work reported in this paper.

## Figures and Tables

**Figure:**
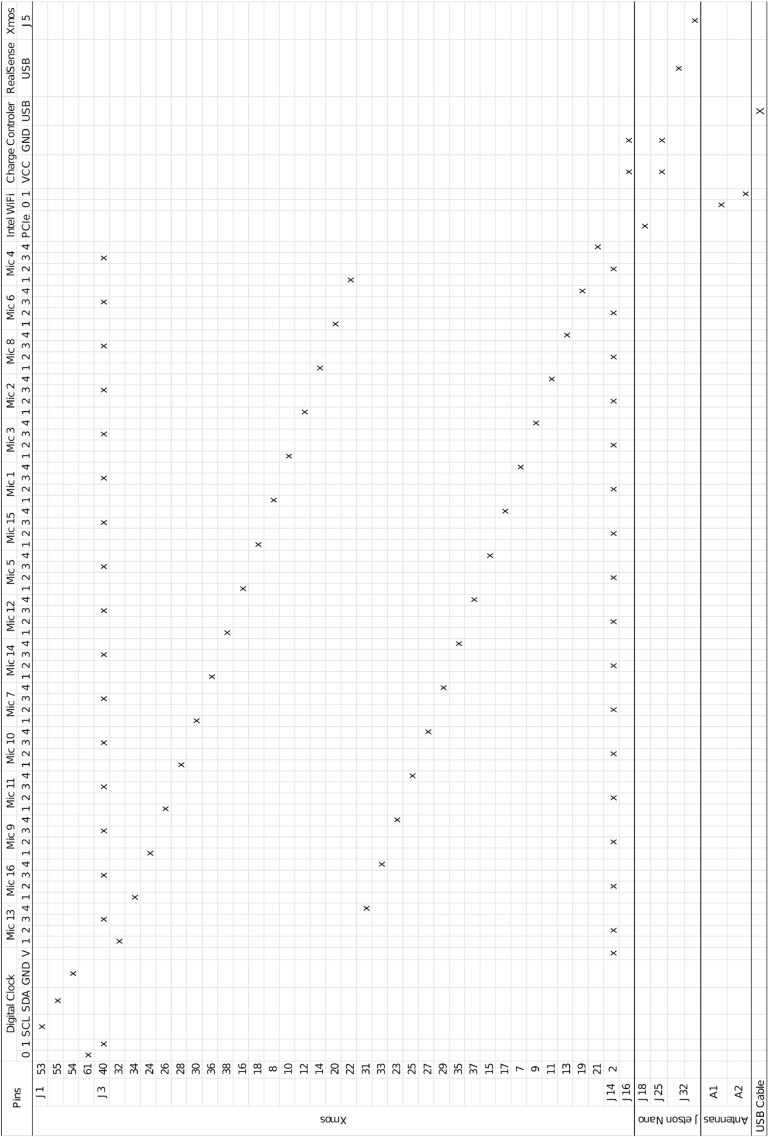


## Footnotes

4 https://www.xmos.com/file/xtimecomposer-community_14-linux64-installer?version=all.

5 https://developer.nvidia.com/embedded/learn/get-started-jetson-nano-devkit.

6 https://jetsonhacks.com/2019/12/22/install-realsense-camera-in-5-minutes-jetson-nano/.

## Footnotes

1 https://www.adafruit.com/product/2045.

2 https://www.adafruit.com/product/5991.

3 https://www.infineon.com/cms/en/product/packages/PG-LLGA/PG-LLGA-5-4/.

7 https://www.arrow.com/en/research-and-events/articles/five-essential-safety-products-for-every-electronics-lab.

8 https://www.dell.com/support/kbdoc/en-us/000137973/safety-precautions-when-working-with-electrical-equipment.

## References

[1] Prüss-Ustün A., Wolf J., Corvalán C., Bos R., Neira M. (2016). https://www.who.int/publications/i/item/9789241565196.

[2] Hahad O., Gilan D., Michal M., Tüscher O., Chalabi J., Schuster A.K., Keller K., Hobohm L., Schmitt V.H., König J., Lackner K.J., Wild P., Schattenberg J.M., Daiber A., Münzel T. (2024). Noise annoyance and cardiovascular disease risk: results from a 10-year follow-up study. Sci. Rep..

[3] Natanael H., Rosmolen M., Grythe J., Prasetiyo I. (2019). Sound power estimation with an acoustic camera in comparison with sound power determination using sound pressure and sound intensity method. J. Phys.: Conf. Ser..

[4] Nelson P., Fahy F., Walker J. (2004). Advanced Applications in Acoustics, Noise and Vibration.

[5] Merino-Martínez R., Sijtsma P., Snellen M., Ahlefeldt T., Antoni J., Bahr C.J., Blacodon D., Ernst D., Finez A., Funke S., Geyer T.F., Haxter S., Herold G., Huang X., Humphreys W.M., Leclère Q., Malgoezar A., Michel U., Padois T., Pereira A., Picard C., Sarradj E., Siller H., Simons D.G., Spehr C. (2019). A review of acoustic imaging methods using phased microphone arrays: Part of the aircraft noise generation and assessment special issue. CEAS Aeronaut. J..

[6] Chiariotti P., Martarelli M., Castellini P. (2019). Acoustic beamforming for noise source localization – reviews, methodology and applications. Mech. Syst. Signal Process..

[7] Eric M.M. (2011). 2011 19thTelecommunications Forum (TELFOR) Proceedings of Papers.

[8] Prime Z., Doolan C. (2013). Proceedings of the Annual Conference of the Australian Acoustical Society.

[9] Infineon Technologies (2022). https://www.infineon.com/cms/en/product/sensor/mems-microphones/mems-microphones-for-consumer/im73d122/.

[10] XMOS (2015). https://www.xmos.com/develop/xcore-200.

[11] Intel Corporation (2024). https://dev.intelrealsense.com/docs/intel-realsense-d400-series-product-family-datasheet.

[12] NVIDIA Corporation (2020). https://developer.nvidia.com/embedded/learn/get-started-jetson-nano-devkit.

[13] Aarts R., Janssen A. (2009). Estimating the velocity profile and acoustical quantities of a harmonically vibrating loudspeaker membrane from on-axis pressure data. J. Audio Eng. Soc..

[14] Liu J., Liu Y., Bolton J.S. (2018). Acoustic source reconstruction and visualization based on acoustic radiation modes. J. Sound Vib..

[15] Yu L., Antoni J., Zhao H., Guo Q., Wang R., Jiang W. (2021). The acoustic inverse problem in the framework of alternating direction method of multipliers. Mech. Syst. Signal Process..

[16] International Organization for Standardization (2010). https://www.iso.org/standard/52055.html.

[17] International Electrotechnical Commission (2014). https://webstore.iec.ch/en/publication/5063.

[18] Wang Z., Wu S.F. (1997). Helmholtz equation–least-squares method for reconstructing the acoustic pressure field. J. Acoust. Soc. Am..

[19] Hansen P.C., O’Leary D.P. (1993). The use of the L-curve in the regularization of discrete ill-posed problems. SIAM J. Sci. Comput..

[20] Ekstrom M.P., Rhoads R.L. (1974). On the application of eigenvector expansions to numerical deconvolution. J. Comput. Phys..

